# Purification of MNase for Use in Ribosomal Profiling of High-Salinity Extremophiles

**DOI:** 10.21769/BioProtoc.5811

**Published:** 2026-09-20

**Authors:** Pavlina Gregorova, Matthew F. Isada, Jocelyne DiRuggiero, Peter L. Sarin

**Affiliations:** 1 RNAcious Laboratory, Department of Molecular and Integrative Biosciences, Faculty of Biological and Environmental Sciences, University of Helsinki, Helsinki, Finland; 2 Department of Biology, Johns Hopkins University, Baltimore, MD, USA

**Keywords:** Micrococcal nuclease, S7, MNase, Ribosome profiling, Haloarchaea, Halophiles, Protein purification

## Abstract

Nucleases are key tools in molecular biology, enabling controlled nucleic acid digestion for applications such as ribosome profiling. Micrococcal nuclease (MNase) from *Staphylococcus aureus* is widely used as a tool in molecular biology and biochemistry, but its reduced activity under high-salt conditions necessitates higher enzyme input to achieve efficient digestion, increasing costs in studies of halophilic organisms. Here, we present an optimized protocol for the heterologous expression and purification of the recombinant staphylococcal MNase. The procedure enables reproducible production of a highly active, stable enzyme and incorporates an enzymatic activity assay to standardize batches to minimize variability. The resulting MNase exhibits robust activity in high-salt environments and remains stable during storage, providing a cost-effective and reliable alternative to commercial nucleases for ribosome profiling and related applications.

Key features

• Expression and one-step purification of MNase for use in ribosome profiling.

• Validation of purified protein by absorbance-based enzymatic activity assay.

## Background

Micrococcal nuclease (MNase), derived from *Staphylococcus aureus* [1], is widely used in molecular biology thanks to its ability to cleave both RNA and DNA in a calcium-dependent manner [2,3]. In *S. aureus*, MNase is produced as a secreted precursor (MNaseB) that is proteolytically processed into a shorter mature form (MNaseA) [4]. Both forms are enzymatically active and have been applied in various biochemical applications such as nucleosome mapping and chromatin accessibility assays [5], protein–nucleic acid interaction studies [6], and ribosome profiling in bacteria [7,8], including the more recent application in halophilic organisms [9]. However, MNase-based applications in halophilic systems face practical limitations. Commercial MNase preparations are often costly and may not provide sufficient activity in high-salt conditions, necessitating increased enzyme input. In addition, existing MNase production protocols predominantly focus on MNaseB [10]. Our previous work has shown that the mature form, MNaseA, exhibits higher activity under high-salt conditions compared to MNaseB, making it more suitable for applications such as ribosome profiling in halophilic organisms [11].

Here, we describe an optimized protocol for the expression and characterization of highly active MNaseA suitable for use in high-salt environments. Compared to existing methods, this approach offers improved enzyme yield, cost efficiency, and reproducibility, particularly for applications such as ribosome profiling in halophilic and prokaryotic systems. Beyond ribosome profiling, the protocol can also support a range of molecular biology applications, including chromatin analysis, RNA processing studies, and workflows requiring efficient nucleic acid degradation under challenging conditions.

## Materials and reagents

*Note: Equivalent materials and reagents may be used as substitutes.*

**Biological materials**

**Figure 1.**
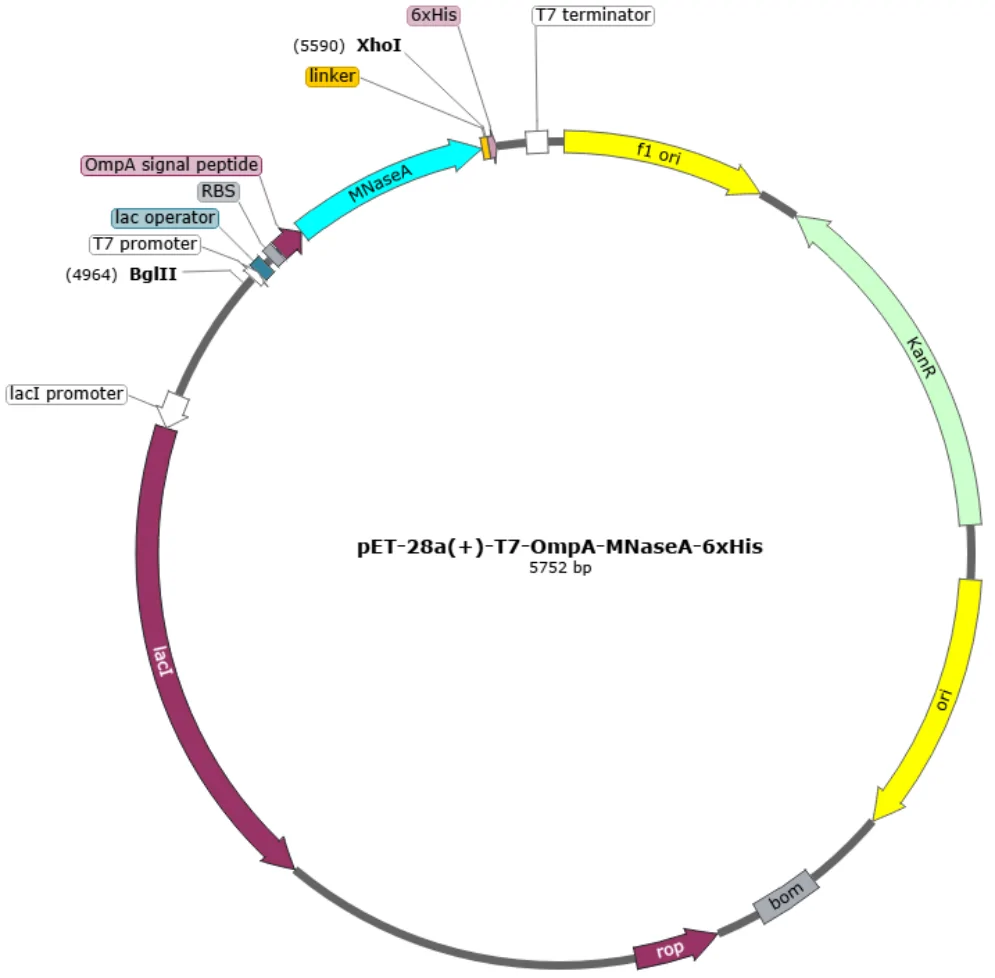
Plasmid map of the MNase expression vector. Modified from [11].

1. Chemi-competent *E. coli* Lemo21(DE3) (NEB, catalog number: C2528J)

2. pET-28a(+)-T7-OmpA-MNaseA-6xHis (Addgene, catalog number: 214808) (see Figure 1)

3. Plasmid or genomic DNA with 2 μg/μL concentration (e.g., *E. coli* gDNA or high copy plasmid such as pUC18/19)

**Reagents**

1. Calcium chloride dihydrate (CaCl_2_·2H_2_O) (Thermo Scientific Chemicals, catalog number: 447325000, CAS: 10035-04-8)

2. Chloramphenicol (Fisher BioReagents, catalog number: BP904100, CAS: 56-75-7)

3. Ethylenediaminetetraacetic acid (EDTA), pure (Thermo Scientific, catalog number: 118432500, CAS: 60-00-4)

4. Glycerol (Fisher Chemicals, catalog number: G/0650/08, CAS: 56-81-5)

5. Glycine (Fisher BioReagents, catalog number: BP3811, CAS: 56-40-6)

6. Halt^TM^ protease inhibitor cocktail, EDTA-free, 100× (Thermo Scientific, catalog number: 87785)

7. HisPur^TM^ Ni-NTA resin (Thermo Scientific, catalog number: 88222)

8. Hydrochloric acid (HCl), 37% (Fisher Chemical, catalog number: H/1150/PB15, CAS: 7647-01-0)

9. Imidazole (Thermo Scientific Chemicals, catalog number: 122025000, CAS: 288-32-4)

10. InstantBlue^®^ Coomassie protein gel stain (Abcam, catalog number: ISB1L)

11. Isopropyl-β-D-thiogalactopyranoside (IPTG), dioxane-free (Thermo Scientific, catalog number: R0392, CAS: 367-93-1)

12. Kanamycin sulfate (Fisher BioReagents, catalog number: BP9065, CAS: 25389-94-0)

13. L-(+)-rhamnose (98+%) (Thermo Scientific Chemicals, catalog number: A16166, CAS: 10030-85-0)

14. Laemmli sample buffer, 4× (Bio-Rad, catalog number: 1610747)

15. NaCl (Fisher Chemical, catalog number: S/3120/63, CAS: 7647-14-5)

16. Prestained or unstained protein ladder (Thermo Scientific, catalog number: 26630 or 26619)

17. Protein Assay Kit II (Bio-Rad, catalog number: 5000002)

18. Sodium hydroxide (NaOH) (Fisher Chemical, catalog number: S/4920/60, CAS: 1310-73-2)

19. Tris base (Fisher BioReagents, catalog number: BP1521, CAS: 77-86-1)

20. Tryptone (Fisher BioReagents, catalog number: BP9726500)

21. Yeast extract (Fisher BioReagents, catalog number: BP1422500)

22. Liquid nitrogen

23. Sodium dodecyl sulphate (SDS) (Fisher BioReagents, catalog number: BP166500, CAS: 151-21-3)

24. SDS-PAGE gels, 15% or 16% (e.g., Thermo Scientific, Novex^TM^ Tris-Glycine Mini Protein Gels, 16%, 1.0 mm, XP00165BOX or equivalent hand-cast gels)

25. (optional) Nuclease S7 (MNase) (Roche, catalog number: 10107921001)

**Solutions**

1. Protein expression

a. 100 mM L-(+)-rhamnose (see Recipes)

b. 1 M IPTG (see recipes)

c. 50 mg/mL kanamycin (Kan) (see Recipes)

d. 35 mg/mL chloramphenicol (Cm) (see Recipes)

e. LB-Luria (see Recipes)

2. Components for purification buffers

a. 1 M imidazole (see Recipes)

b. 1 M Tris pH 7.5 (see Recipes)

c. 5 M NaCl (see Recipes)

d. 1 M CaCl_2_ (see Recipes)

e. 0.5 M EDTA (see Recipes)

f. 80% (v/v) glycerol (see Recipes)

3. MNase purification buffers

a. Lysis buffer (see Recipes)

b. Wash buffer (see Recipes)

c. Elution buffer (see Recipes)

d. Storage buffer (see Recipes)

4. Enzymatic activity assay

a. 100 mM CaCl_2_ (see Recipes)

b. 1 M Tris-HCl, pH 8.0 (see Recipes)

c. 100 mM Tris-HCl, pH 8.0 (see Recipes)

d. 10 mM Tris-HCl, pH 8.0 (see Recipes)

5. 10× Tris-Glycine-SDS running buffer (see Recipes)

**Recipes**

*Notes:*

*1. When purifying the enzyme, calculate the amount of buffer needed (especially for the lysis buffer due to the cost of the Halt protease inhibitor). We also tested purification without protease inhibitors in the buffers and found that using an inhibitor only in the lysis buffer is sufficient to retain high protein quality.*

*2. The following recipes are the approximate amounts for MNase purification from 1 L of* E. coli *culture.*

**1. Protein expression**

**a. 100 mM L-(+)-rhamnose**

**Table:** 

Reagent	Final concentration	Quantity or volume
L-(+)-rhamnose	100 mM	0.18 g
Ultra-pure water	n/a	up to 10 mL
Total	n/a	10 mL

Filter-sterilize (0.2 μm) and store aliquots at -20 °C for up to 1–2 years. Avoid repeated freeze-thaw cycles.

**b. 1 M IPTG**

**Table:** 

Reagent	Final concentration	Quantity or volume
IPTG	1 M	2.38 g
Ultra-pure water	n/a	up to 10 mL
Total	n/a	10 mL

Filter-sterilize (0.2 μm) and store aliquots at -20 °C for up to 1 year. For reproducible MNase expression, it is crucial to use the recommended brand of IPTG powder listed above.

**c. 50 mg/mL Kan**

**Table:** 

Reagent	Final concentration	Quantity or volume
Kanamycin sulfate	50 mg/mL	0.5 g
Ultra-pure water	n/a	up to 10 mL
Total	n/a	10 mL

Dissolve and filter-sterilize (0.2 μm). Store aliquots at -20 °C for up to 1 year. Avoid repeated freeze-thaw cycles.

**d. 35 mg/mL Cm**

**Table:** 

Reagent	Final concentration	Quantity or volume
Chloramphenicol	35 mg/mL	0.35 g
99.9% ethanol	99.9%	10 mL
Total	n/a	10 mL

Dissolve and store aliquots at -20 °C for up to 1 year. Avoid repeated freeze-thaw cycles.

**e. LB-Luria**

**Table:** 

Reagent	Final concentration	Quantity or volume
NaCl	0.5 g/L	0.5 g
Yeast extract	5 g/L	5 g
Tryptone	10 g/L	10 g
Ultra-pure water	n/a	up to 1 L
Total	n/a	1 L

Dissolve all components, adjust to the final volume, and filter-sterilize (0.2 μm). Store at room temperature.

*Note: We noticed that the MNase processing during expression in* E. coli *Lemo21(DE3) is improved when the medium is filter sterilized (0.2 μm) rather than autoclaved.*

**2. Components for purification buffers**

**a. 1 M imidazole**

**Table:** 

Reagent	Final concentration	Quantity or volume
Imidazole	1 M	6.8 g
Ultra-pure water	n/a	up to 100 mL
Total	n/a	100 mL

Dissolve and filter-sterilize (0.2 μm). Store at 4 °C in a dark bottle for up to 2 years.

**b. 1 M Tris-HCl, pH 7.5**

**Table:** 

Reagent	Final concentration	Quantity or volume
Tris base	1 M	12.11 g
Ultra-pure water	n/a	up to 100 mL
Total	n/a	100 mL

Dissolve Tris base in ~70 mL of ultra-pure water. Once dissolved, adjust pH to 7.5 by adding HCl (concentrated or slightly diluted). Fill to final volume with ultra-pure water and filter-sterilize (0.2 μm). Store at room temperature for up to 1 year.

**c. 5 M NaCl**

**Table:** 

Reagent	Final concentration	Quantity or volume
NaCl	5 M	29.22 g
Ultra-pure water	n/a	up to 100 mL
Total	n/a	100 mL

Dissolve and filter-sterilize (0.2 μm). Store at room temperature indefinitely.

**d. 1 M CaCl_2_**

**Table:** 

Reagent	Final concentration	Quantity or volume
CaCl_2_·2H_2_O	1 M	14.70 g
Ultra-pure water	n/a	up to 100 mL
Total	n/a	100 mL

Dissolve and filter-sterilize (0.2 μm). Store at room temperature indefinitely.

**e. 0.5 M EDTA**

**Table:** 

Reagent	Final concentration	Quantity or volume
EDTA	0.5 M	14.61 g
Ultra-pure water	n/a	up to 100 mL
Total	n/a	100 mL

EDTA does not dissolve below pH 8.0. To prepare a 0.5 M EDTA solution, add the weighted amount of EDTA to ~70 mL of ultra-pure water and dissolve it by slowly adding either NaOH pellets (approximately 3–4 g/100 mL) or 5 M NaOH solution. Fill to the final volume with ultra-pure water and filter-sterilize (0.2 μm). Store at room temperature for several years.

**f. 80% (v/v) glycerol**

**Table:** 

Reagent	Final concentration	Quantity or volume
Glycerol	80%	80 mL
Ultra-pure water	n/a	up to 100 mL
Total	n/a	100 mL

Autoclave the solution and store it at room temperature for several years.

**3. MNase purification buffers**

*Note: All purification buffers can be prepared one day in advance (without protein inhibitors) and kept at 4 °C. Never use buffers older than 1 week old. Always add inhibitors (100× Halt protease inhibitor, etc.) immediately prior to use.*

**a. Lysis buffer**

**Table:** 

Reagent	Final concentration	Volume
1 M CaCl_2_	1 mM	0.050 mL
5 M NaCl	250 mM	2.5 mL
1 M Tris-HCl, pH 7.5	50 mM	2.5 mL
1 M imidazole	5 mM	0.250 mL
80% (v/v) glycerol	5% (v/v)	3.125 mL
100× Halt protease inhibitor	0.5×	0.250 mL
Ultra-pure water	n/a	up to 50 mL
Total	n/a	50 mL

Prepare freshly just before using. Store at 4 °C. Add 100× Halt inhibitor just before use.

**b. Wash buffer**

**Table:** 

Reagent	Final concentration	Volume
5 M NaCl	250 mM	5 mL
1 M Tris-HCl, pH 7.5	50 mM	5 mL
1 M imidazole	20 mM	2 mL
80% glycerol	5% (v/v)	6.25 mL
Ultra-pure water	n/a	up to 100 mL
Total	n/a	100 mL

Store at 4 °C for up to 1 week.

**c. Elution buffer**

**Table:** 

Reagent	Final concentration	Volume
5 NaCl	250 mM	2.5 mL
1 M Tris-HCl, pH 7.5	50 mM	2.5 mL
1 M imidazole	250 mM	12.5 mL
80% (v/v) glycerol	5% (v/v)	3.125 mL
Ultra-pure water	n/a	up to 50 mL
Total	n/a	50 mL

Store at 4 °C for up to 1 week.

**d. Storage buffer**

**Table:** 

Reagent	Final concentration	Volume
5 M NaCl	50 mM	1 mL
0.5 M EDTA	1 mM	0.2 mL
1 M Tris-HCl, pH 7.5	50 mM	5 mL
80% glycerol	5%	6.25 mL
Ultra-pure water	n/a	up to 100 mL
Total	n/a	100 mL

Store at 4 °C for up to 1 week.

**4. Enzymatic activity assay**

**a. 100 mM CaCl_2_**

Dilute from 1 M CaCl_2_ by mixing 5 mL of 1 M solution and 45 mL of sterile ultra-pure water. Store at room temperature for up to 1 year.

**b. 1 M Tris-HCl, pH 8.0**

**Table:** 

Reagent	Final concentration	Quantity or volume
Tris base	1 M	12.11 g
Ultra-pure water	n/a	up to 100 mL
Total	n/a	100 mL

Dissolve Tris base in ~70 mL of ultra-pure water. Once dissolved, adjust pH to 8.0 by adding HCl (concentrated or slightly diluted). Fill to the final volume with ultra-pure water and filter-sterilize (0.2 μm). Store at room temperature for up to 1 year.

**c. 100 mM Tris-HCl, pH 8.0 (50 mL)**

Dilute from 1 M Tris-HCl, pH 8.0, by mixing 5 mL of 1 M solution and 45 mL of sterile ultra-pure water. Store at room temperature for up to 1 year.

**d. 10 mM Tris-HCl, pH 8.0 (50 mL)**

Dilute from 100 mM Tris-HCl, pH 8.0, by mixing 5 mL of 1 M solution and 45 mL of sterile ultra-pure water. Store at room temperature for up to 1 year.

**5. 10× Tris-Glycine-SDS running buffer**

**Table:** 

Reagent	Final concentration	Quantity or volume
Tris base	0.25 M	30.3 g
Glycine	1.92 M	144 g
SDS	1%	10 g
Ultra-pure water	n/a	Up to 1 L
Total	n/a	1 L

Prepare a 10× stock solution by dissolving the components in ~800 mL of ultra-pure water. Once dissolved, adjust to the final volume. Prepare 1× working buffer before use by diluting with ultra-pure water.

**Laboratory supplies**

1. 1.5 mL microcentrifuge tubes (Fisher Scientific, catalog number: 11926955 or equivalent)

2. 50 mL screw-cap centrifuge tubes (Sarstedt, catalog number: 62.547.254 or equivalent)

3. 15 mL screw-cap centrifuge tubes (Sarstedt, catalog number: 62.554.001 or equivalent)

4. 0.5–10 μL pipette tips (Fisher Scientific, catalog number: 11913426 or equivalent)

5. 5–300 μL pipette tips (Fisher Scientific, catalog number: 11903456 or equivalent)

6. 100–1,250 μL pipette tips (Fisher Scientific, catalog number: 10492725 or equivalent)

7. 25 mL serological pipettes (Sarstedt, catalog number: 86.1685.001 or equivalent)

8. 10 mL serological pipettes (Sarstedt, catalog number: 86.1254.001 or equivalent)

9. 96-well UV-transparent microplates (Corning, catalog number: 3635)

10. Pierce^TM^ centrifuge columns (Thermo Scientific, catalog number: 89898)

11. Amicon^®^ Ultra Centrifugal Filter, 10 kDa MWCO (Merck Millipore, catalog number: UFC901024)

12. Nunc 96-well microplate (Thermo Scientific, catalog number: 260836, or equivalent compatible with colorimetric measurements)

13. Bottle top or syringe filters, 0.2 μm (Fisher Scientific, catalog number: 15973307 or 15206869 or equivalent)

14. Disposable cuvettes semi-micro (VWR, catalog number: 634-0676)

15. Cultivation flasks for *E. coli* culturing, e.g., 100 mL, 250 mL, and 5 L (any)

16. Petri dishes (any)

17. Sterile inoculation loops (any)

18. Glass bottles, beakers, and volumetric flasks for buffer and media preparation (any)

## Equipment

1. Tube revolver rotator (Thermo Scientific, catalog number: 88881001)

2. Cryogenic mixer mill or sonicator (Retsch, model: MM400; or Hielscher Ultrasonics, model: UP400S)

3. Multiscan FC microplate photometer with wavelength 450/595 nm (ThermoFisher Scientific, catalog number: 1410101)

4. Superspeed Centrifuge Sorvall, model LYNX 4000 (Thermo Scientific, catalog number: 75008590)

5. Fiberlite F12-6 × 500 fixed angle rotor (Thermo Scientific, catalog number: 096-062375)

6. Centrifugation bottles for F12 rotor (Thermo Scientific, catalog number: 3141-0500PK)

7. Temperature-controlled UV-plate reader (PerkinElmer, model: EnSpire or equivalent)

8. Stripettor^®^ Pro Pipet Controller (Corning, catalog number: 4999 or equivalent)

9. Adjustable volume pipettes 1–10 μL (Fisher Scientific, catalog number: 11835762 or equivalent)

10. Adjustable volume pipettes 10–100 μL (Fisher Scientific, catalog number: 11865762 or equivalent)

11. Adjustable volume pipettes 100–1,000 μL (Fisher Scientific, catalog number: 11885762 or equivalent)

12. Variable volume, multichannel pipettes 10–100 μL (Fisher Scientific, catalog number: 11825772 or equivalent)

13. Protein gel apparatus, e.g., Mini-PROTEAN^®^ Tetra Vertical Electrophoresis (Bio-Rad, catalog number: 1658004 or equivalent)

14. Power supply, PowerPac^TM^ HC High-Current Power Supply (Bio-Rad, catalog number: 1645052 or equivalent)

15. Incubator shaker New Brunswick^TM^ Excella E25 Shaker (Eppendorf or equivalent)

16. Incubator 37 °C (any)

17. Centrifuge 5427 R with rotor FA-45-24-11 (1.5/2 mL tubes) (Eppendorf, catalog number: 5429000010 or equivalent)

18. Centrifuge 5810 R with swing-bucket rotor A-4-62 (Eppendorf, catalog number: 5811000015 or equivalent)

19. Open Air Rocker (Fisher Scientific, catalog number: 88861026 or equivalent)

20. QB Series Dry Block Heating Systems (Grant, model: QBD2 or equivalent)

21. UV-Vis spectrophotometer (any)

22. 744 pH Meter (Metrohm, model: 744 or equivalent)

23. Direct 8 Milli-Q Direct Water Purification System with filter Biopak Polisher (Merck, catalog number: CDUFBI001)

24. Balance AB104-S/PH (Mettler Toledo, catalog number: 11135020 or equivalent)

25. Diaphragm Vacuum Pump LABOPORT (KNF, type: N 810.3 FT.18 or equivalent for filter sterilization)

26. NanoDrop 2000c Spectrophotometer (Thermo Scientific, catalog number: ND-2000C or equivalent)

27. Vortexer (any)

28. Magnetic hotplate stirrer, e.g., Isotemp RT Advanced Hotplate Stirrer (Fisher Scientific, catalog number: 15326607 or equivalent)

## Software and datasets

1. Prism (GraphPad Software, Version 11.0.0, requires license)

2. Excel (Microsoft Office 365, requires license, or other spreadsheet software)

## Procedure

**A. MNase expression**

*Note: Always use a freshly transformed plate. Do not use >1-week-old transformations. We recommend not using glycerol stocks of transformed clones.*

1. Inoculate 20–50 mL of LB-Luria broth containing 50 μg/mL Kan and 35 μg/mL Cm with a single colony of *E. coli* Lemo21(DE3) transformed with plasmid pET-28a(+)-T7-OmpA-MNaseA-6xHis (Figure 1).

2. Grow overnight (<16 h) at 37 °C, 200 rpm.

3. Next day, measure OD_600_ of the overnight culture.

4. Prepare expression culture containing 50 μg/mL Kan, 35 μg/mL Cm, and 500 μM L-rhamnose.

a. Add 1 mL of 50 mg/mL Kan, 1 mL of 35 mg/mL Cm, and 5 mL of 100 mM L-rhamnose to 1 L of LB-Luria broth in a 5 L cultivation flask.

*Note: For cultivation, choose the correct flask size. The best results are obtained when using a maximum fill volume of 20% (e.g., 1 L of culture in a 5-L flask).*

b. Inoculate the culture with *E. coli* starter to OD_600_ = 0.1.

*Note: Users can also prepare the main culture by 100× diluting the overnight starter culture (e.g., 10 mL of* E. coli *starter for 1 L of LB-Luria broth). Although both approaches work well, inoculation to OD_600_ = 0.1 might result in faster growth.*

c. Grow at 37 °C, 200 rpm until OD_600_ = 0.4–0.5.

*Note: Growth takes typically 2–3 h.*

**Critical**: If the culture is grown in a 5-L flask, use 160 rpm instead of 200 rpm.

5. While the culture is growing, turn on another incubator and prewarm it to 30 °C.

6. Once the cells reach OD_600_ = 0.4–0.5, take 180 μL of the uninduced control sample for SDS-PAGE analysis.

a. Collect cells by centrifugation at 13,000× *g* for 1 min at 4 °C.

b. Transfer the supernatant to a new 1.5 mL tube and mix with 60 μL of 4× SDS loading dye.

c. Resuspend the pellet in 30 μL of 1× SDS loading dye (loading dye diluted from 4× in ultra-pure water).

d. Boil both samples for 10 min at 95 °C.

e. Store samples at -20 °C if not analyzed on the same day.

7. Move cultures to a 30 °C shaker and induce MNase expression by adding 1 mL of 1 M IPTG per 1 L of culture (final concentration 1 mM).

8. Express protein at 30 °C, 200 rpm, for 8–10 h.

*Note: When cultivating smaller culture volumes than 1 L, the peak expression and MNase processing are typically achieved within 8 h. For 1-L cultures, longer expression (at least 10 h) is required to obtain the maximum amount of processed MNase.*

9. At the end of the expression, collect 90 μL of culture (induced control) for SDS-PAGE analysis and process the sample as described in step A6. Add 30 μL of 4× SDS loading dye to the supernatant.

10. Collect the expression cell culture by centrifugation at 4,000–5,000× *g* for 15 min at 4 °C. Discard supernatant.

**Critical:** We tested two lysis methods—sonication and cryogenic milling—that can be applied during MNase purification. Depending on the method used, the harvested cells need to be prepared and stored differently. Both methods yield similar results.

11. Scrape the cell pellets into weighed, pre-cooled 50 mL tubes (in liquid nitrogen or dry ice).

12. Snap-freeze the pellets in liquid nitrogen and weigh the tubes again. Record the weight of each pellet.

**Pause point:** Cell pellets can be stored at -80 °C for <1 week. However, for the most optimal results, process the pellets immediately the following day.

13. **Alternative procedure** for preparation of harvested cells for lysis by cryogenic milling

*Note: This procedure describes cryogenic milling of harvested cells using a Retsch MM400 mixer mill with 50 mL vials (maximum load ~10–15 g per vial). In this approach, milling efficiency is improved when cells are processed as frozen droplets rather than compact pellets. Therefore, cell pellets are first resuspended in lysis buffer, and the resulting suspension is sprayed directly into liquid nitrogen to form droplets. The lysis buffer volume used here is optimized for 50 mL vials of the Retsch MM400. If larger cryogenic mills are available (e.g., SPEX SamplePrep 6870 Freezer/Mill), the buffer volume can be increased to improve pellet resuspension. Adjust the volume according to the capacity of your equipment. For milling with SPEX 6870, use 8 CPS, 5 cycles (1 min on, 1 min off) for ~7.5–9.3 g per sample during cryomilling (each sample derived from ~500 mL of culture). For this instrument, the sample remains submerged in liquid nitrogen throughout the entire cryomilling process. These conditions resulted in incomplete cell lysis but still provided substantial protein yield.*

a. Scrape the cell pellets (from step A10) into weighed, pre-cooled 50 mL tubes (on ice).

b. Resuspend the harvested cell pellets in 2.5–3 mL of lysis buffer per 1 g of wet cell pellet. Keep the suspension on ice.

*Note: This is the most labor-intensive step. As it is time-consuming, keep cells on ice or perform the procedure in a cold room.*

**Figure 2.**
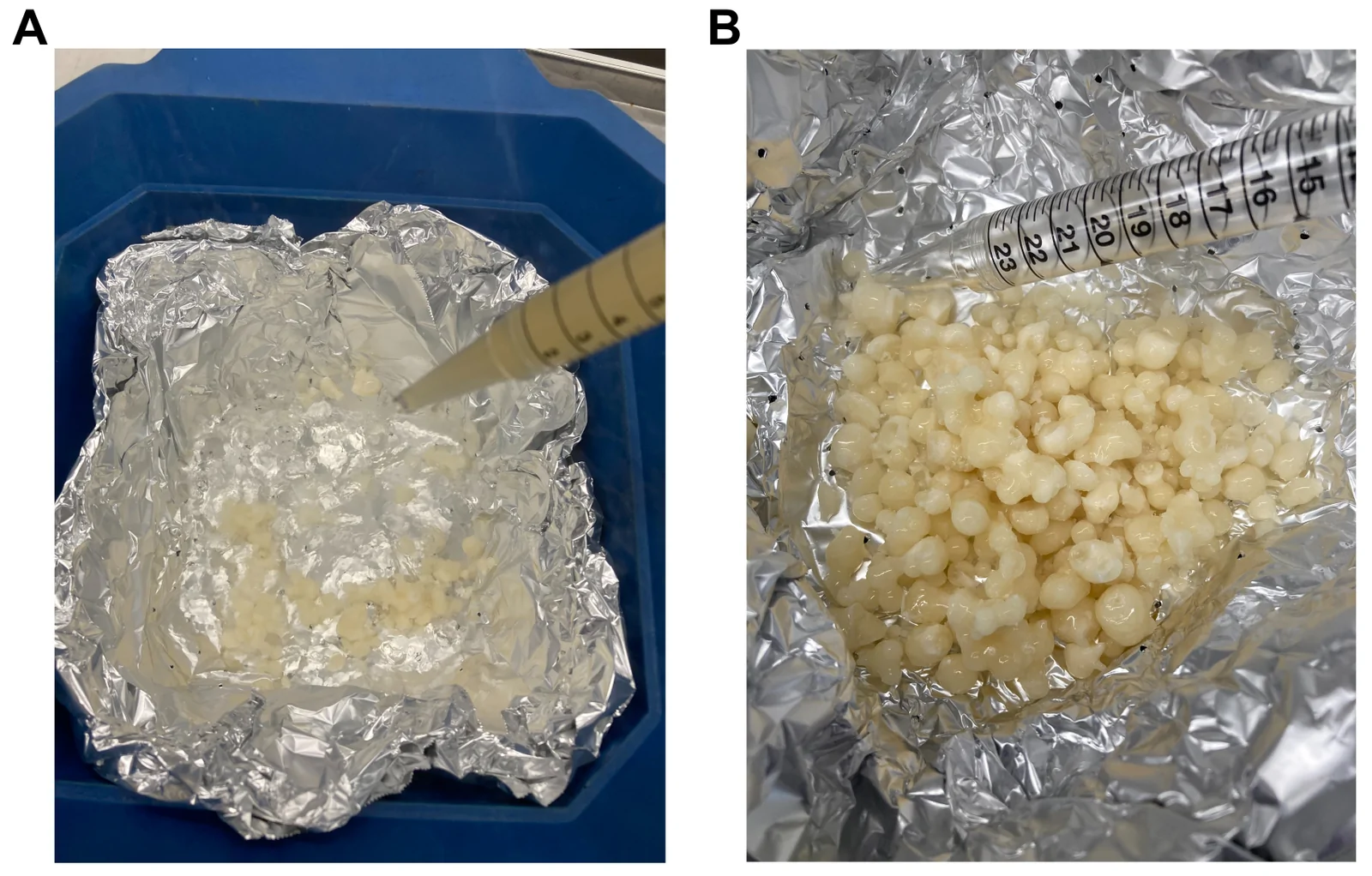
Liquid nitrogen bath used for freezing expression cultures. (A) Bath is made from aluminum foil perforated with small holes using a needle and placed in a standard ice bucket. Liquid nitrogen is added, and the culture is sprayed directly into the bath. (B) Cell droplets are harvested by lifting the foil from the ice bucket, allowing liquid nitrogen to drain away.

c. Set up a liquid nitrogen bath to prepare cell suspension droplets.

i. Line the ice bucket with 2–3 layers of aluminum foil.

ii. Puncture small holes in the bottom of the foil using a needle.

iii. Fill the bucket halfway with liquid nitrogen.

d. Prepare frozen droplets of cell suspension by spraying cell suspension directly into the liquid nitrogen bath using a serological pipette (Figure 2).

e. Collect frozen droplets into 50 mL tubes.

i. Lift the foil out of the liquid nitrogen bath.

ii. Allow excess liquid nitrogen to drain through the holes in the foil.

iii. Transfer frozen droplets into pre-cooled 50-mL tubes kept in liquid nitrogen or on dry ice.

iv. Store tubes at -80 °C or directly proceed with cryogenic milling.

**Pause point:** Cell suspension droplets can be stored at -80 °C for <1 week.

**B. MNase purification**

*Notes:*

**Figure 3.**
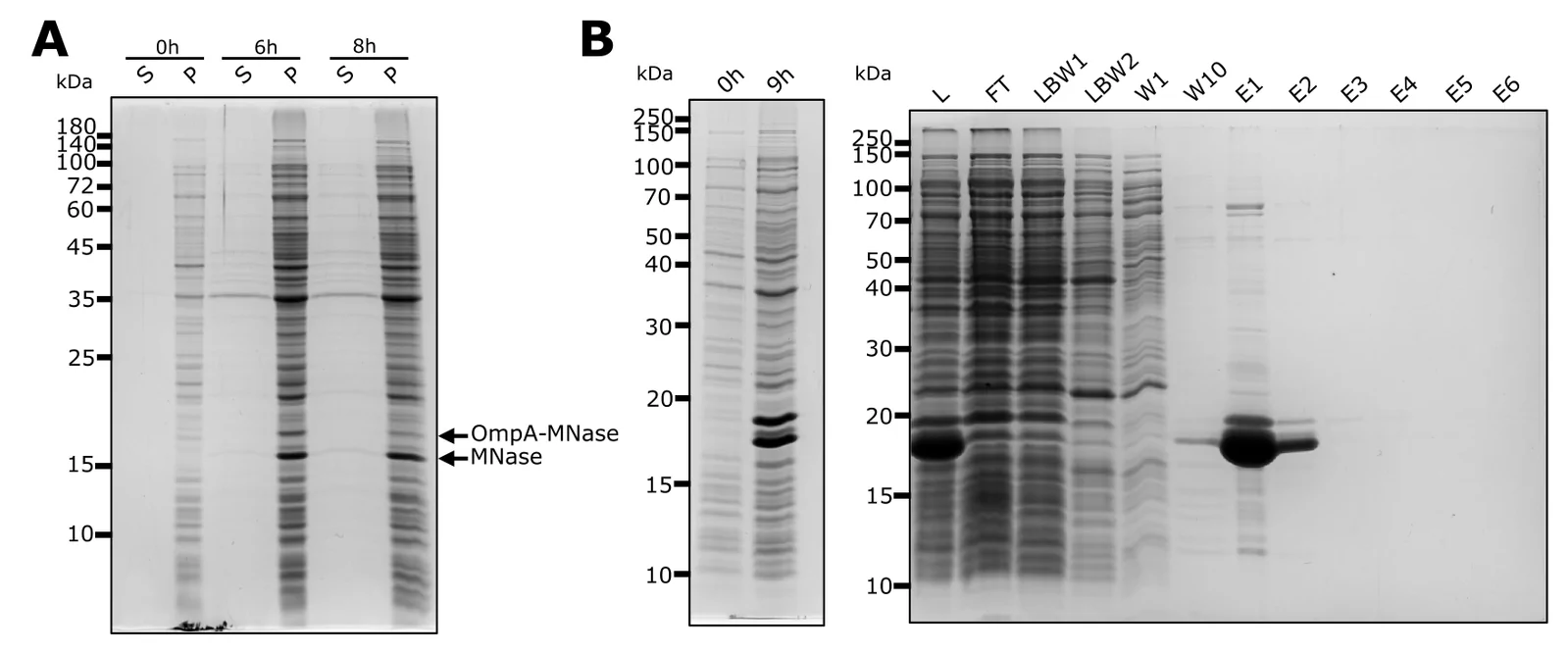
MNase expression and purification. (A) MNase expression in 200 mL of *E. coli* Lemo21(DE3) culture and cleavage of the OmpA signal sequence from MNase increases over time. S, supernatant; P, pellet. (B) Example of SDS-PAGE analysis of samples collected during MNase purification from 1 L culture. L, lysate; FT, flowthrough; LBW, lysis buffer wash; W, wash; E, elution.

*1. Prior to purification, verify MNase expression (see Figure 3A) by analyzing uninduced and induced samples collected in steps A6 and A9 by SDS-PAGE gel.*

*2. The MNase purification from 1 L of expression culture typically yields 11–17 mg of pure enzyme.*

1. Lyse cells by sonication

a. Melt harvested cells on ice for 5 min.

b. Add 5 mL of cold lysis buffer per 1 g of wet cell pellet and resuspend cells by vortexing or pipetting.

*Note: If the suspension is too viscous, add more lysis buffer and, for reproducibility, note down the total amount.*

c. Using the smallest sonicator probe, lyse the cells by sonication on ice for 10 cycles, 60% amplitude, 0.5 cycle, 20 s ON/1 min OFF.

**Critical:** Choose a sonication tip appropriate to the amount of lysate. Using a bigger tip can lead to lysate foaming and overheating, which may result in decreased MNase activity.

*Note: The sonication time may vary depending on instrument model and should be optimized as needed. Due to MNase nuclease activity, the lysate will not become viscous, unlike typical cell lysates.*

d. After sonication, mix the lysate by inverting the tube.

e. Clarify lysate by centrifugation at 10,000× *g* for 30 min at 4 °C.

f. Keep clarified lysate on ice and collect 9 μL of clarified lysate sample for SDS-PAGE analysis. Process sample similarly to other protein gel samples (see section C). Proceed immediately to step B3.

g. **Alternative procedure** for cell lysis by cryogenic milling

*Note: This is an example of a protocol that was successfully used to cryogenically prepare lysates using the Retsch MM400 mixer mill. Keep in mind that an alternative milling program might be needed for different models of cryogenic mills.*

**Caution:** Ensure you are familiar with the manufacturer’s instructions and always follow the manual when operating the cryogenic mill.

i. Pre-cool a 50 mL milling jar containing a single 10 mm bead in liquid nitrogen.

ii. Transfer frozen cell suspension droplets to the milling jar.

iii. Cool the jar with frozen cell suspension for 1 min in liquid nitrogen.

iv. Attach the jar to the mill and mill the cells at 30 Hz, 1.5 min ON followed by 1 min OFF. During OFF time, remove the jar from the mill and immerse it in liquid nitrogen.

v. Perform six cycles and inspect the vial content. If the material is grinded to powder, proceed with the next steps. If not, perform additional milling cycles (up to 10 in total).

**Critical:** Ensure cells are fully pulverized into fine powder. If in doubt, we recommend performing an additional milling rather than proceeding with an insufficiently milled sample. Insufficiently milled cells will lead to reduced MNase yields.

vi. Transfer the powder from the milling jar to a 50 mL tube.

vii. Let the cell lysate thaw on ice.

viii. Add more lysis buffer. Use the same ratio as in step B1b.

ix. Mix the thawed lysate by inverting the tube.

x. Clarify the lysate by centrifugation at 10,000× *g* for 30 min at 4 °C.

xi. Keep the clarified lysate on ice and collect 9 μL of lysate sample for SDS-PAGE analysis. Process samples as other gel samples (see section C). Proceed immediately to step B3.

2. Prepare Ni-NTA resin

*Note: Perform these steps during step B1e (clarification of lysate).*

a. Mix the Ni-NTA resin slurry thoroughly by inversion.

b. Transfer 5–6 mL of Ni-NTA resin slurry to a 15 mL tube.

*Note: Use 5–6 mL of 50% slurry/1 L pellet. Scale down proportionally for smaller culture volumes.*

c. Centrifuge the tube at 700× *g* for 2 min; then, carefully remove and discard the supernatant.

d. Add two resin-bed volumes (2.5–3 mL) of cold lysis buffer, then mix by inversion until the resin is fully suspended.

e. Centrifuge the tube at 700× *g* for 2 min, then carefully remove and discard the buffer.

f. Repeat steps B2d–e at least once more (ideally 2×).

3. Purify MNase.

**Critical:** Perform the whole purification procedure in a 4 °C room.

a. Incubate the clarified lysate with washed Ni-NTA beads (see step B2) at 4 °C, rotating for 1–2 h.

b. After incubation, load the beads with supernatant into the Pierce centrifuge column or similar self-packable gravity flow column.

*Note: To recover all beads from the tube, rinse with additional lysis buffer, which will be used for the first wash (see step B3d).*

c. Collect the flowthrough (FT) into a sterile bottle or 50 mL tube.

d. Wash with two column volumes (CV) of lysis buffer. Collect the lysis buffer wash (LBW) fraction separately into a new tube.

*Note: Use the Ni-NTA bead volume to define the CV, e.g., 3 mL of 50% Ni-NTA bead slurry means that the CV will be 3 mL.*

e. Wash the column with 10 CVs of wash buffer, collecting the first and last wash fractions separately. Store fractions at 4 °C.

f. Elute MNase with 6 CVs of elution buffer and collect each elution fraction separately. Store fractions at 4 °C.

**Critical:** Do not store elution fractions at 4 °C for longer than overnight due to the high imidazole concentration. Proceed with the next steps as soon as possible, as imidazole is harmful to the enzyme.

g. Analyze all collected samples on a 15% or 16% SDS-PAGE gel. For a detailed list of samples, see section C and Table 1.

h. Stain the gel with InstantBlue^®^ Coomassie protein gel stain.

i. Pool the desired elution fractions (typically the first 2–3 fractions; see Figure 3B) containing MNase.

j. Concentrate MNase and perform buffer exchange into storage buffer using a 10 kDa MWCO centrifugal concentrator.

k. Centrifuge at 4,000× *g* (swing-bucket rotor) for 20–30 min at 4 °C or until the volume decreases to ~1–2 mL.

l. Directly to the concentrator, add 8–9 mL of storage buffer and repeat step B3k.

m. Repeat steps B3l at least 4–5 times.

*Note: The number of buffer exchange steps required to reduce the imidazole concentration below 1 mM can be calculated. An example is provided in Supplementary Table 1.*

**Critical:** Never interrupt the concentration/buffer exchange procedure. If the buffer exchange procedure is not complete and MNase is left on ice for longer periods of time, protein precipitation is likely to occur.

m. After buffer exchange, concentrate MNase to ~1 mL when purifying from a 1 L culture.

**Critical:** Do not concentrate MNase below 1 mL to avoid precipitation.

n. Transfer the MNase from the protein concentrator to a clean 2 mL tube. Collect also a small sample (10–20 μL) for protein quantification (see section C) and the enzymatic activity assay (see section D).

o. Measure the protein concentration of purified MNase using Protein Assay Kit II or another equivalent protein quantification assay.

p. Prepare 30 μL of MNase at 33.3 ng/μL. Add 10 μL of 4× SDS loading dye to obtain a final concentration of 25 ng/μL. Boil both samples for 10 min at 95 °C. Store at -20 °C unless analyzed immediately.

q. Aliquot the remaining concentrated MNase into 50- or 100-μL aliquots and flash-freeze them in liquid nitrogen.

**Pause point:** The protein can be stored at -80 °C for at least 1 year. Furthermore, the concentrated MNase withstands 4–5 freeze cycles if stored at -80 °C.

r. Analyze the final MNase on a protein SDS-PAGE gel to assess its purity (see section C).

**C. Analysis of protein samples**

*Note: This section describes how protein samples can be prepared and analyzed by SDS–PAGE. An example of gel analysis is shown in Figure 3.*

1. Unless otherwise indicated (see Table 1), pellet all collected protein samples at 13,000× *g* for 1 min.

2. Mix the supernatant with 4× SDS loading dye in a 3:1 ratio and resuspend the pellet in 1× SDS loading dye (see Table 1).

3. Boil samples at 95–100 °C for 10 min.

*Note: If not analyzed immediately, store samples at -20 °C. Before loading, boil samples again for 5 min.*

**Table 1.** Protein sample collection and analysis

Sample	Collected volume	Loading dye volume*	Loaded volume to gel
Uninduced cell culture (pellet)	-	30 μL	3 μL
Uninduced cell culture (supernatant)	180 μL	30 μL	12 μL
Induced cell culture (pellet)	-	30 μL	3 μL
Induced cell culture (supernatant)	90 μL	30 μL	12 μL
Clarified cell lysate	9 μL	3 μL	3 μL
Flowthrough (FT)	90 μL	30 μL	12 μL
Lysis buffer wash (LBW)	90 μL	30 μL	12 μL
Wash (W)	90 μL	30 μL	12 μL
Elution (E)	3 μL	1 μL	1 μL

Use 4× SDS loading dye for supernatants/liquid samples and 1× SDS loading dye for cell pellets.

4. Gel loading

*Note: The gel analysis is qualitative only, as equal loading by cell or culture amount is difficult to achieve.*

a. Load samples according to Table 1.

b. Load 4, 8, and 20 μL of purified MNase diluted to 25 ng/μL. These volumes correspond to 100, 200, and 500 ng, respectively.

**D. MNase activity assay**

*Notes:*

*1. This assay is performed under optimal MNase activity conditions and is intended for batch-to-batch comparison of purified MNase. A commercial MNase (see Materials and reagents) may be included as a reference.*

*2. Prepare enough DNA substrate (plasmid or genomic DNA) to allow consistent use across multiple MNase batches. Plasmid DNA stores well at -20 °C.*

1. Dilute the MNase to a concentration of 0.01 μg/μL using 10 mM Tris-HCl, pH 8.0. Keep the enzyme dilution on ice.

2. Prepare the substrate as described in Table 2 and store it on ice.

**Table 2.** Reaction mix for activity assay

Component	Final concentration	Per 1 reaction	Master mix (30×)
100 mM Tris-HCl, pH 8.0	10 mM	5 μL	150 μL
100 mM CaCl_2_	10 mM	5 μL	150 μL
2 μg/μL DNA (substrate)	0.2 μg/μL	5 μL	150 μL
Ultra-pure water	n/a	35 μL	1050 μL

3. Turn on the plate reader and pre-heat it to 25 °C. Load the analysis program. The plate reader should be ready to load samples and start analysis immediately after adding substrate to the diluted enzyme (see next steps).

4. Place the UV-compatible plate on ice.

*Note: To prevent condensation on the bottom of the plate, place a paper tissue and a plastic sheet between the ice and the plate. This keeps the plate cold while minimizing condensation.*

5. Prepare enzyme dilutions in quadruplicate directly in the plate, as described in Table 3.

**Table 3.** MNase dilutions for activity assay

Row on plate	Volume of MNase	Volume of 10 mM Tris-HCl, pH 8.0	MNase amount (μg/reaction)
B (blank)	0 μL	50 μL	0 μg
C	3 μL	47 μL	0.03 μg
D	6 μL	44 μL	0.06 μg
E	12 μL	38 μL	0.12 μg
F	24 μL	26 μL	0.24 μg
G	48 μL	2 μL	0.48 μg

6. Add 50 μL of substrate to each enzyme dilution. Mix well by pipetting up and down.

7. Measure absorbance at 260 nm every 30 s for 1 h at 25 °C.

## Data analysis

1. Export the absorbance data to csv.

2. Import data to GraphPad Prism. Use an XY table with four replicates.

3. Plot the data points as a function of absorbance (y-axis) with time (x-axis) and perform exponential curve fitting with one-phase association according to the following formula:

$$A_{x}=A_{0}+\left(P-A_{0}\right)\times (1-e^{\left(-K\times x\right)})$$

where

A_x_: absorption A_260_ at time x;

A_0_: absorption A_260_ at time 0;

P: plateau = absorption A_260_ at infinite time;

K: rate constant as reciprocal time;

x: time

*Note: An example is provided in Figure 2 from [11].*

3. Calculate MNase activity from initial slopes for time 0 and 1 using the formula above. The K and P values are found by curve fitting in GraphPad Prism. The calculation of slopes can be done in Excel or other spreadsheet software.

*Note: One unit (U) is defined as an increase of 0.005 A_260_ per 1 min.*

4. Plot MNase activity (y-axis) vs. MNase amount (x-axis) and perform linear regression. The slope corresponds to specific activity (U/μg).

*Note: For specific activity calculation, use only activity values from MNase concentrations that show a linear response.*

5. (Optional) Calculate activity as units per microliter by multiplying the specific activity by the concentration of the MNase stock.

*Note: An example of the analysis is provided in Supplementary Table 2.*

## Validation of protocol

This protocol (or parts of it) has been used and validated in the following research article(s):

• Gregorova et al. [11]. Purification of micrococcal nuclease for use in ribosomal profiling of high-salinity extremophiles. *Journal of Biological Chemistry* (Figures 1–3).

## General notes and troubleshooting

**General notes**

This protocol was optimized for expression in *E. coli* Lemo21(DE3) cells, but other strains [e.g., *E. coli* BL21(DE3)] can be used. Because MNase is expressed with an OmpA signal sequence, tighter regulation improves processing to the mature form; however, retention of the signal sequence does not significantly reduce its enzymatic activity. Moreover, the OmpA-containing form tends to precipitate under the applied buffer conditions and does not bind to Ni-NTA resin, thereby enriching for the correctly processed protein. Lastly, culture scaling may be limited, and optimal results are typically obtained from cultures ≤500 mL.

**Troubleshooting**

**Problem 1:** Low expression or processing of MNase.

Possible causes: Old IPTG stock, old rhamnose stock, or problems with scaling from a smaller to a larger culture.

Solutions: Prepare fresh IPTG and rhamnose. Increase expression time to >10 h.

**Problem 2:** Low yields of purified MNase.

Possible cause: Incomplete cell lysis.

Solutions: Increase the number of sonication cycles or milling cycles. Increasing the lysis buffer volume can also improve lysis.

**Problem 3:** MNase has precipitated.

Possible cause: MNase was concentrated to too high a concentration during buffer exchange.

Solution: Do not concentrate below 1 mL. This can be achieved by shortening the centrifugation steps during buffer exchange and the final concentration step. If precipitation occurs, remove the precipitate by centrifugation at 10,000× *g* for 10 min at 4 °C. Retain the supernatant containing the active MNase.

## Supplementary information

The following supporting information can be downloaded here:

1. Supplementary Table 1. Example of buffer exchange steps calculation

2. Supplementary Table 2. Example of MNase activity calculation
